# High-intensity interval training is an effective exercise mode to maintain normal blood pressure during pregnancy: a randomized control trial

**DOI:** 10.1038/s41598-024-79552-3

**Published:** 2024-11-14

**Authors:** Junjiang Sun, Łukasz Radzimiński, Rita Santos-Rocha, Anna Szumilewicz

**Affiliations:** 1 Department of Fitness, Gdansk University of Physical Education and Sport, 80-336 Gdansk, Poland; 2 Higher Vocational College, Yunnan College of Business Management, Kunming, 650000 China; 3 Department of Physiology, Gdansk University of Physical Education and Sport, 80-336 Gdansk, Poland; 4 ESDRM Sport Sciences School of Rio Maior, Santarém Polytechnic University, 2040-413 Rio Maior, Portugal; 5 SPRINT Sport Physical Activity and Health Research and Innovation Center, 2040-413 Rio Maior, Portugal

**Keywords:** Pregnancy, HIIT, Blood pressure, Cardiopulmonary fitness, Exercise, Physiology, Health care

## Abstract

Pregnant women are more susceptible to high blood pressure (BP) than the general adult population; therefore, all means of preventing this condition should be considered. High-intensity interval training (HIIT) is effective in this regard in the general population, but there is a lack of evidence of its effectiveness during pregnancy. This study aimed to compare an 8-week HIIT program to self-performed moderate-to-vigorous physical activity among pregnant women by evaluating changes in BP after a maximal progressive cardiorespiratory exercise test (CPET) performed at pre-intervention and post-intervention time points. A total of 54 Caucasian women in uncomplicated, singleton pregnancies (age 32 ± 4 years, 22 ± 4 weeks of gestation; M ± SD) with normal BP values completed the interventions. The experimental (HIIT) group (*n* = 34) completed an online supervised HIIT program consisting of three sessions per week and supplemented by an educational class once per week. Participants in the education (EDU) group (*n* = 20) attended an educational class once per week and were encouraged to perform moderate-to-vigorous physical activity (PA) on their own. Pre- and post-intervention, all women underwent a CPET on a cycle ergometer with a respiratory gas analyzer. On the day of the CPET, maternal systolic and diastolic BP (mmHg) was measured at rest (before the CPET) and approximately 60 min after the CPET using an electronic BP monitor. Identical CPET and BP measurement protocols were employed for both the HIIT and EDU groups at the pre- and post-intervention time points. Pre-intervention, the HIIT and EDU groups both showed a decrease in systolic and diastolic BP after the CPET, though only the change in systolic BP was statistically significant (HIIT group: *p* = 0.01; EDU group: *p* = 0.001). Post-intervention, there were no significant differences in either group between resting and post-CPET BP. There were significant post-intervention differences in VO_2peak_ (*p* < 0.001) and HR_max_ (*p* = 0.002) between the HIIT and EDU groups. From pre- to post-intervention, the EDU and HIIT groups both showed decreases in resting systolic or diastolic BP; there was a significant difference in systolic BP in the EDU group (*p* = 0.005) and a significant difference in diastolic BP in the HIIT group (*p* = 0.03). Both groups maintained normal BP values throughout the experiment. However, HIIT, in addition to maintaining normotension, improved cardiorespiratory fitness in pregnant women. It seems that both supervised HIIT and self-performed moderate-to-vigorous PA can be recommended as strategies to prevent BP disorders during pregnancy. More studies are needed to confirm our findings.

*Trial registration* The full study protocol was registered in ClinicalTrials.gov (NCT05009433).

**Supplementary Information:**

The online version contains supplementary material available at 10.1038/s41598-024-79552-3.

## Introduction

During pregnancy, the female body undergoes several adaptations to enable adequate fetal growth^1^. Substantial changes in cardiovascular physiology occur, including a significant increase in blood volume, resting heart rate (HR), and cardiac output as well as a decrease in systemic vascular resistance^2^. Some authors have observed that in healthy pregnancies, blood pressure (BP) decreases during early pregnancy, reaching a nadir in mid-pregnancy before progressively increasing from the second trimester until delivery, after which it returns to pre-pregnancy levels during the early postpartum period^3^. The average increase in BP observed between mid-pregnancy and delivery has been reported to be approximately 8% ^4^. However, other authors found in their recent meta-analysis that the commonly taught mid-pregnancy decrease in BP was not observed in a group of more than 36,000 pregnant women^5^. A normotensive pregnancy is considered to be one in which BP remains below 130/80 mmHg throughout the pregnancy^6^.

Pregnant women are more susceptible to high BP than the general adult population due to alterations in endocrine function, plasma flow increment, and stress levels over the course of the pregnancy^7^. Hypertensive disorders of pregnancy affect approximately 10% of pregnant women globally and are among the leading causes of pregnancy-related mortality and morbidity among women, adolescent girls, and their newborns, particularly in low- and middle-income countries^8–10^. Hypertension during pregnancy can be subdivided into the categories of chronic hypertension, gestational hypertension, and the spectrum of preeclampsia^11^, which may be an indication of preeclampsia when arising around or after 20 weeks of gestation^8^. Early antihypertensive treatment and timely delivery can prevent morbidity and potentially mortality in cases of preeclampsia^12^. Improving the management of hypertension during pregnancy is, therefore an essential aspect of quality care for maternal and neonatal health^13^. On the other hand, during the second time of hormonal shifts, rapid changes in the maternal circulatory system can cause hypotension, which affects approximately 10% of pregnant women^14^. Hypotension during pregnancy (BP < 110/65 mmHg) may cause dizziness or faintness, which may inhibit participation in physical activity (PA) and lead to falls and potential fetal and maternal trauma^15,16^. Patients presenting with low BP should be asked standardized questions, including questions regarding their symptoms (e.g., fatigue, dizziness, nausea, lightheadedness, thermoregulation in warm environments, etc.), the time of their last meal or snack, their water intake, and their BP history, to gain insight into their current health and determine if hypotension is a common occurrence or preexisting condition^16^.

Exercise may help to normalize BP levels and cardiovascular conditioning^17^ and reduce the incidence of hypertension^18^ in pregnant women. It may protect against preeclampsia by reducing maternal concentrations of oxidative substances (oxidative stress), stimulating vascularity and placental growth, and preventing endothelial dysfunction^19^. Exercise additionally ensures adequate venous blood flow to the heart, preventing drops in BP^20^ and hypotensive symptoms. The current evidence of the positive impact of physical exercise on BP during pregnancy primarily relates to moderate-to-vigorous exercise, which is the standard exercise mode recommended for pregnant women^21^.

High-intensity interval training (HIIT) has been gaining popularity as an effective exercise strategy in recent years. HIIT is a contemporary protocol that was developed for non-athletes with the goals of reducing session time and providing a greater stimulus for physiological and psychological adaptation^22^. The effectiveness of HIIT in preventing or treating numerous health conditions, including hypertension, has been proven in various populations^23^. However, there is limited evidence of its effects in pregnant women^24^. Currently, the efficacy of HIIT programs in regulating BP during pregnancy is unknown. Therefore, the primary objective of this study was to compare an 8-week HIIT program to independently performed moderate PA among pregnant women by evaluating changes in BP after a maximal exercise test at pre-intervention and post-intervention time points.

## Materials and methods

### Participants

Sixty-nine Caucasian women in uncomplicated, singleton pregnancies (age 32 ± 4 years, 22 ± 4 weeks of gestation) were invited to participate in the study. Participants were randomly allocated by the principal researcher to either the experimental (HIIT) or the comparison education (EDU) group at a 1:1 ratio. Both interventions had a duration of 8 weeks.

The eligibility criteria for participation were as follows: (1) course of pregnancy allowing for participation in PA adapted for pregnant women; (2) gestational week no later than 28 weeks to enable completion of the intervention before childbirth; (3) proficiency in the Polish language; and (4) age of 18 to 45 years. The exclusion criteria were as follows: (1) any contraindications to physical exertion or other conditions that could adversely affect the health or safety of the participant or the quality of the collected data; (2) multiple pregnancies; and (3) lack of a tablet or computer with Internet access. We used the list of contraindications to increased physical exertion during pregnancy in line with the Get Active Questionnaire in Pregnancy^25^. After receiving a thorough explanation of all procedures and the potential risks involved, all participants provided written informed consent before the baseline testing and interventions were initiated. All participants received routine obstetric care throughout the trial in accordance with national law. In addition, participants were asked to follow the recommendations for a healthy diet for pregnant women while enrolled in the study.

**Fig. 1:**
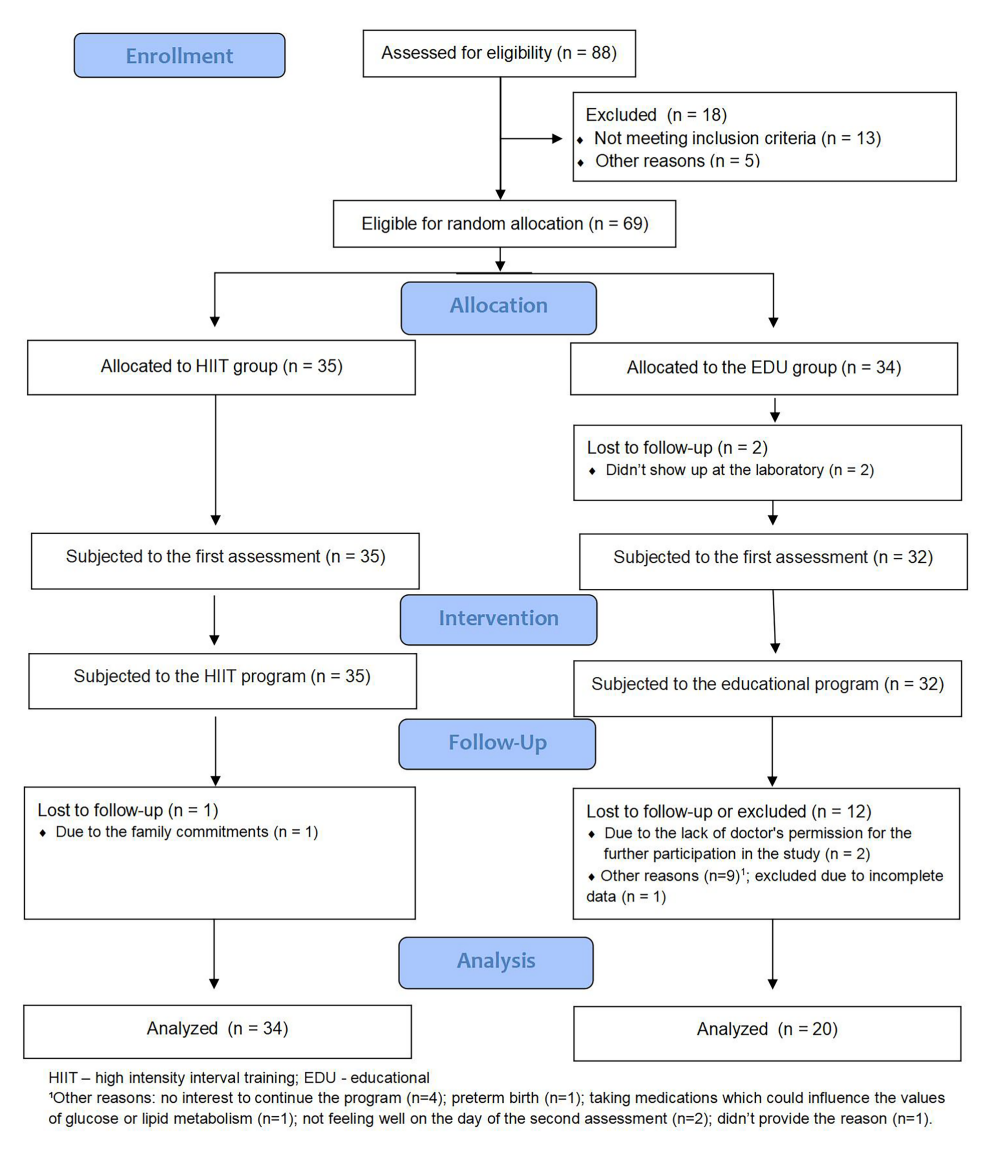
Flowchart of participants through the study.

Initially, 35 pregnant women were randomly assigned to the HIIT group. One participant attended only three sessions before withdrawing from the intervention due to family responsibilities. Thirty-four pregnant women were assigned to the EDU group to participate in an 8-week educational program. Twelve women from this group were lost to follow-up or excluded from the analysis. The final analysis thus included 54 pregnant women: 34 in the HIIT group and 20 in the EDU group. Figure 1 presents a flowchart of the participants throughout the study.

The study was conducted in the Laboratory of Physical Effort and Genetics in Sport at the Gdansk University of Physical Education and Sport in Poland in 2021. It was performed according to the principles of the WMA Declaration of Helsinki and with the approval of the Bioethics Commission of the District Medical Chamber in Gdansk (KB − 8/21). All participants gave written informed consent before beginning the study. The exercise program was delivered by a qualified exercise professional. The participation was for fee, and pregnant women were informed that they would be free to drop-out the study anytime without any consequences. The full study protocol was registered in ClinicalTrials.gov (NCT05009433) on 17/08/2021. No important methodological changes were made after trial commencement. The standards for transparency, openness, and reproducibility of research were followed throughout the study^26^.

### Study variables and instrumentation

**Fig. 2:**
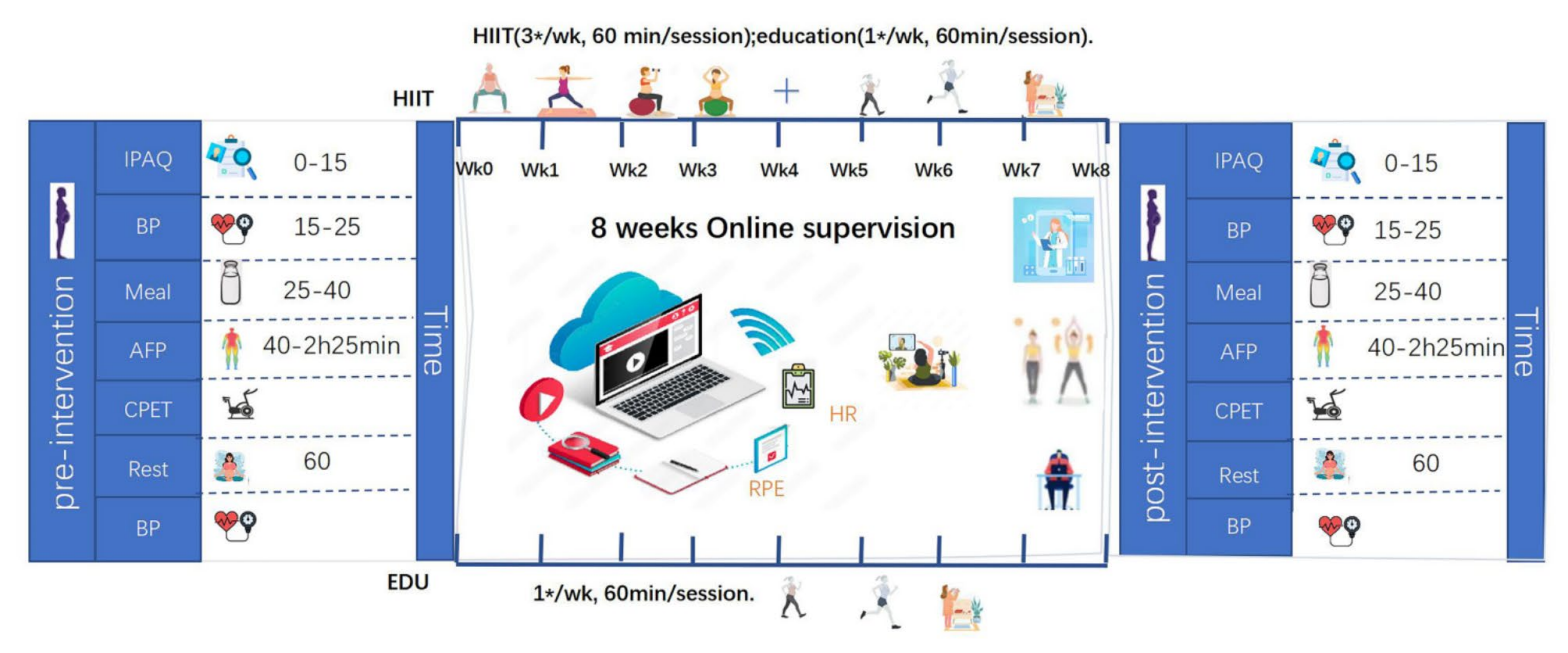
Schematic representation of the study protocol. EDU: education, HIIT: high-intensity interval training, HR: heart rate, IPAQ: International Physical Activity Questionnaire, BP: blood pressure, AFP: assessment of functional parameters, CPET: cardiorespiratory maximal progressive exercise test, RPE: Rating of Perceived Exertion.

The measurement and intervention processes employed during the study are shown in Fig. 2. Data was collected before the initiation and after the completion of the 8-week HIIT and educational programs.

#### Characteristics of study participants and physical activity levels

Demographic data (i.e., age, education level, occupation), height, weight, and information regarding participants’ health and pregnancy course were collected before the start of the interventions. PA level was assessed using the short version of the International Physical Activity Questionnaire (IPAQ)^27^. This questionnaire has been shown to have acceptable measurement characteristics and provides information on weekly PA levels in multiples of resting metabolic rate. The participants were classified into one of three PA levels based on their IPAQ results: low (inactive), medium (accumulation of PA at the minimum recommended level), or high (PA above the minimum recommended level)^28,29^.

#### Cardiorespiratory maximal progressive exercise test and blood pressure measurement

A cardiorespiratory maximal progressive exercise test (CPET) was conducted according to American Thoracic Society/American College of Chest Physicians recommendations^30^ using a cycle ergometer with an electronically regulated load (Viasprint 150P; Bitz, Germany) and a pulmonary gas analyzer (Oxycon Pro; Erich Jaeger GmbH, Hoechberg, Germany). The ergometer was calibrated according to the manufacturer’s instructions. Breath-by-breath data were averaged to elicit a data point for each 15-second period. Before the test, the participants sat in a chair while wearing the silicon mask for 5 min to acclimate their breathing. After the acclimation period, the participants completed a warm-up by cycling at a relative load of 0.4 W·kg^− 1^ for 4 min. When the warm-up was finished, the load was increased by 0.2 W·kg^− 1^ per minute until participants indicated that they could no longer continue. Before the test began, the participants were encouraged to cycle to the limit of their physical ability and informed that they could stop the test at any time. After completing the test, participants took a 3-minute seated break.

On the day of the CPET, maternal systolic and diastolic BP (mmHg) were measured at rest (before the CPET) and approximately 60 min after the CPET with participants in a seated position using an electronic BP monitor (OMRON M5 Professional; Mannheim, Germany). Identical CPET and BP measurement protocols were employed for the HIIT and EDU groups at both time points, before and after the 8-week interventions. The wattage applied during the CPET was calculated based on the current weight of the participant; thus, during post-intervention testing, the wattage was adjusted for participants’ weight gain.

BP refers to the pressure of circulating blood against the blood vessel walls and is usually expressed as the systolic pressure (maximum pressure during one heartbeat) over the diastolic pressure (minimum pressure between two heartbeats) during the cardiac cycle^31^. In this study, normal BP was defined as resting BP values ≤ 130/80 mmHg^6,32^. If BP values were below 110/65 mmHg, additional questions were asked to identify hypotensive participants^16^. All participants in this study were classified as normotensive during baseline measurements.

The following additional cardiopulmonary parameters were measured for further analysis: (1) HR at rest; (2) maximum HR (HR_max_), which was the highest number of beats per minute recorded during the CPET; and (3) the maximum oxygen consumption recorded and maintained for 15 s during the CPET (VO_2peak_).

### Experimental training and educational interventions

The HIIT intervention consisted of three 60-minute training sessions per week for 8 weeks. A warm-up and instruction on how to perform the workout lasted 7–10 min. The primary training part of the sessions comprised 15–20 min of high-intensity intervals, which consisted of exercises involving major muscle groups (e.g., squats, lunges, little hops and skips combined with upper body exercises). Individual intervals lasted 30–60 s and were alternated with 30–60 s of rest at a 1:2, 1:1, or 2:1 ratio of exercise time to rest time, depending on the individual ability of the participant and taking into account the progression of training and stage of pregnancy. Following the interval training component, participants performed resistance, postural, neuromotor (e.g., balance), and stretching exercises for 5–10 min. The final cool-down part included pelvic floor muscle and birth preparation exercises, such as birth positions and breathing exercises (5–10 min), as well as relaxation and pregnancy and birth visualization exercises (5–15 min). No equipment was needed to complete the program, as all exercises utilized only body weight resistance. The exercise program was accessible to all participants regardless of their health, exercise capacity, and motor skill level; based on diagnostic exercise testing, the exercise program was tailored to each woman’s individual needs and abilities^24,33,34^.

Based on the outcomes of the CPET, exercise HR target values were calculated for each participant. All participants were instructed to use HR monitors (Polar RS400; Finland) and train at intensities that exceeded their anaerobic threshold HR value (HR/AT) for as long as they felt comfortable during the training intervals. HR/AT values were determined utilizing a modified V-slope method and the ventilatory equivalent method^35^ and were an average of 87% ± 5% of participants’ HR_max_. Individual HR values from all sessions were recorded for further analysis. Although not all participants were able to exercise at intensities above their HR/AT during all intervals, the intervention still qualified as a HIIT program. According to the definition by Wood et al., HIIT protocols should be based on short work intervals (< 60 s–8 min) of vigorous intensity (70–90% of HR_max_ or 14–16 on the 6–20-point Borg Rating of Perceived Exertion [RPE] scale) to high intensity (≥ 90% HR_max_ or ≥ 17 on the 6–20 RPE) interspersed with active (40–70% HR_max_ or 8–13 on the 6–20 RPE) or passive (cessation of movement) recovery periods of 1–5 min^22^.

The study participants were instructed in the 0–10-point Borg RPE scale^36^ and the talk test^37^ as additional measures of exercise intensity. After each session, participants completed individual Exercise Monitoring Cards, where they reported the date of the exercise session, the form of PA performed (participants were asked to enter all forms of PA, including individual activity such as walking or cycling), exercise duration, subjective assessment of exercise intensity per the 0–10 RPE, rest time after exercise, well-being during and after exercise, the reason for any absence from a HIIT session, and any additional comments, which could be important for the data analysis^38^.

HIIT training sessions were conducted online using the MS Teams^®^ platform every Monday, Wednesday, and Friday from 9:30 AM to 10:30 AM, with only one Monday excluded due to being a holiday (23 sessions in total). Participants attended an average of 19 ± 4 sessions (80% of the total training program). Before beginning the program, participants were trained in the use of the MS Teams^®^ application and instructed on safety rules for exercising at home, including proper organization of the exercise space and communication protocols in the event of an accident or deteriorating health condition. The HIIT intervention was complemented by weekly educational sessions. These educational sessions were conducted by the principal researcher, who was qualified as a Pregnancy and Postnatal Exercise Specialist according to European educational standards^24^. Email and telephone contact were utilized to monitor compliance with the program.

The EDU group consisted of 20 pregnant women who attended educational sessions on healthy lifestyles, perinatal PA, and other topics relating to pregnancy and motherhood. The education program was the same as that provided to the HIIT group. The educational sessions were conducted online in real time once per week for 8 weeks. Participants in the EDU group were encouraged to exercise independently, following the recommendations of at least 150 min of moderate-to-vigorous activity per week^34^ (Fig. 2). The EDU group recorded any PA on Exercise Monitoring Cards, including structured exercise sessions and daily activities lasting at least 10 min, such as cleaning the house, gardening, and shopping. The EDU group did not monitor exercise intensity with an HR monitor but instead used the 0–10 RPE scale and talk test. The target exercise intensity was the level at which participants felt a significant increase in respiratory rate but did not feel that their breathing interfered with their ability to converse. On average, participants in the EDU group reported 19 PA sessions with an average intensity of 6 ± 1 on the 0–10 RPE scale.

Throughout the study, all participants remained under standard obstetric care. None of the interventions had a negative impact on participants’ pregnancy process or delivery parameters. Because the trial implementation required the HIIT group to participate in three exercise sessions per week and the EDU group to receive one educational session per week, it was not possible to blind the study participants to group assignment. However, the researchers collecting data were blinded to participants’ group assignments.

### Statistical analysis

Statistical tests were performed using the IBM Statistical Package for the Social Sciences version 25.0 (IBM Corporation; Armonk, New York, USA), and statistical significance was set at *p* ≤ 0.05. The chi-square test was used to compare the non-parametric demographic characteristics between the EDU and HIIT groups. Most variables were expressed as means ± standard deviations, and the analysis of the normality of the distribution of study variables was performed using the Shapiro-Wilk test. The Student’s t-tests were used to determine between-group differences in selected variables at baseline. We used repeated measure ANOVA to analyze the changes in systolic BP and diastolic BP within and between HIIT and EDU groups along with the time of the measurements: before and after CPET, and pre and post the 8-week interventions. The partial eta- squared (η^2^) was used to indicate effect size (effect size conventions: small, η^2^ ≥ 0.01; medium, η^2^ ≥ 0.06 ; large, η^2^ ≥ 0.14). In case of variables with a distribution significantly different from the normal distribution we used non-parametric testing with Friedman test. In order to estimate effect size we used Kendall’s W for testing with Friedman test. Additionally, to better understand the effectiveness of individual PA interventions and to analyze them separately, we used paired-samples T-test to determine within-group differences before and after CPET, pre and post the 8-week interventions. In cases where the distribution was significantly different from the normal distribution, the nonparametric Wilcoxon t-test was performed to assess within-group differences. Pearson’s correlation coefficients were employed to analyze the relationship between BP and weight, parity, body mass index (BMI), and cardiorespiratory fitness (HR, HR_max_, and VO_2peak_) in the HIIT and EDU groups.

The sample size was predetermined by using a power calculation of the test estimating the differences in maximal cardiopulmonary exercise test between HIIT and EDU group after 8-week of intervention. The estimated values of the mean and SD from preliminary tests with 9 women form the HIIT and 9 from the EDU group allowed us to predetermine the minimal sample size of each group 18 with a power of 0.9 and alpha of 0.05.

## Results

### Characteristics of study participants

The characteristics of the study participants are presented in Table 1. The groups did not differ significantly in age, trimester of pregnancy, BMI, education, or PA level. There were significantly more nulliparous women in the EDU group than in the HIIT group (*p* = 0.02).

Before the 8-week interventions, there were no statistically significant differences between the HIIT and EDU groups in any of the cardiopulmonary parameters. The mean systolic BP at rest was 113.31 ± 10.45 mmHg in the entire sample, 111.76 ± 9.42 mmHg in the HIIT group, and 115.95 ± 11.77 mmHg in the EDU group. The mean diastolic BP at rest was 73.06 ± 7.36 mmHg in the entire sample, 73.12 ± 6.63 mmHg in the HIIT group, and 72.95 ± 8.66 mmHg in the EDU group. Systolic and diastolic BP in both groups were within the normal ranges for pregnancy.

**Table 1:** Characteristics of study participants before the 8-week interventions.

Variable		Group (M ± SD or *n* [%])			*p*
		All participants (*N* = 54)	HIIT (*N* = 34)	EDU (*N* = 20)	
Age (years)		34 ± 4	31 ± 4	32 ± 4	0.504^#^
BMI (kg/m^2^)		24.79 ± 2.92	24.44 ± 2.75	25.38 ± 3.18	0.259^#^
Education	Higher	49 (90.7%)	31 (91.2%)	18 (90.0%)	0.885^##^
	Secondary	5 (9.3%)	3 (8.8%)	2 (10.0%)	
Trimester	13–27 weeks (second trimester)	50 (92.6%)	31 (91.2%)	19 (95.0%)	0.604^##^
	28 weeks (third trimester)	4 (7.4%)	3 (8.8%)	1 (5.0%)	
Number of childbirths	0	36 (66.7)	18 (52.9%)	18 (90.0%)	0.02^##^*
	1	10 (18.5%)	9 (26.5%)	1 (5.0%)	
	≥ 2	8 (14.8%)	7 (20.6%)	1 (5.0%)	
Physical activity level	Low	10 (18.5%)	6 (17.6%)	4 (20.0%)	0.829^##^
	Moderate	25 (46.3%)	15 (44.1%)	10 (30.0%)	
	High	19 (35.2%)	13 (38.2%)	6 (50.0%)	
Systolic BP at rest (mmHg)		113.31 ± 10.45	111.76 ± 9.42	115.95 ± 11.77	0.157^#^
Diastolic BP at rest (mmHg)		73.06 ± 7.36	73.12 ± 6.63	72.95 ± 8.66	0.937^#^
HR at rest (beat min^− 1^)		84.11 ± 12.67	82.00 ± 10.11	87.70 ± 15.77	0.111^#^
HR_max_ (beat min^− 1^)		166.85 ± 10.95	167.32 ± 10.07	166.05 ± 12.54	0.684^#^
VO_2peak_ (mL/kg/min)		24.96 ± 4.13	25.71 ± 4.29	23.69 ± 3.58	0.082^#^

BMI: body mass index, EDU: education, HIIT: high-intensity interval training, PA: physical activity, HR: heart rate, VO_2peak_: maximal oxygen uptake, BP: blood pressure, M ± SD: mean ± standard deviation; N: number, p: level of statistical significance, * *p* < 0.05. The analysis was conducted using independent Student’s t-test (#) and the chi-square test (##).

### Analysis of blood pressure parameters

#### Blood pressure before and after cardiorespiratory maximal progressive exercise test

**Fig. 3:**
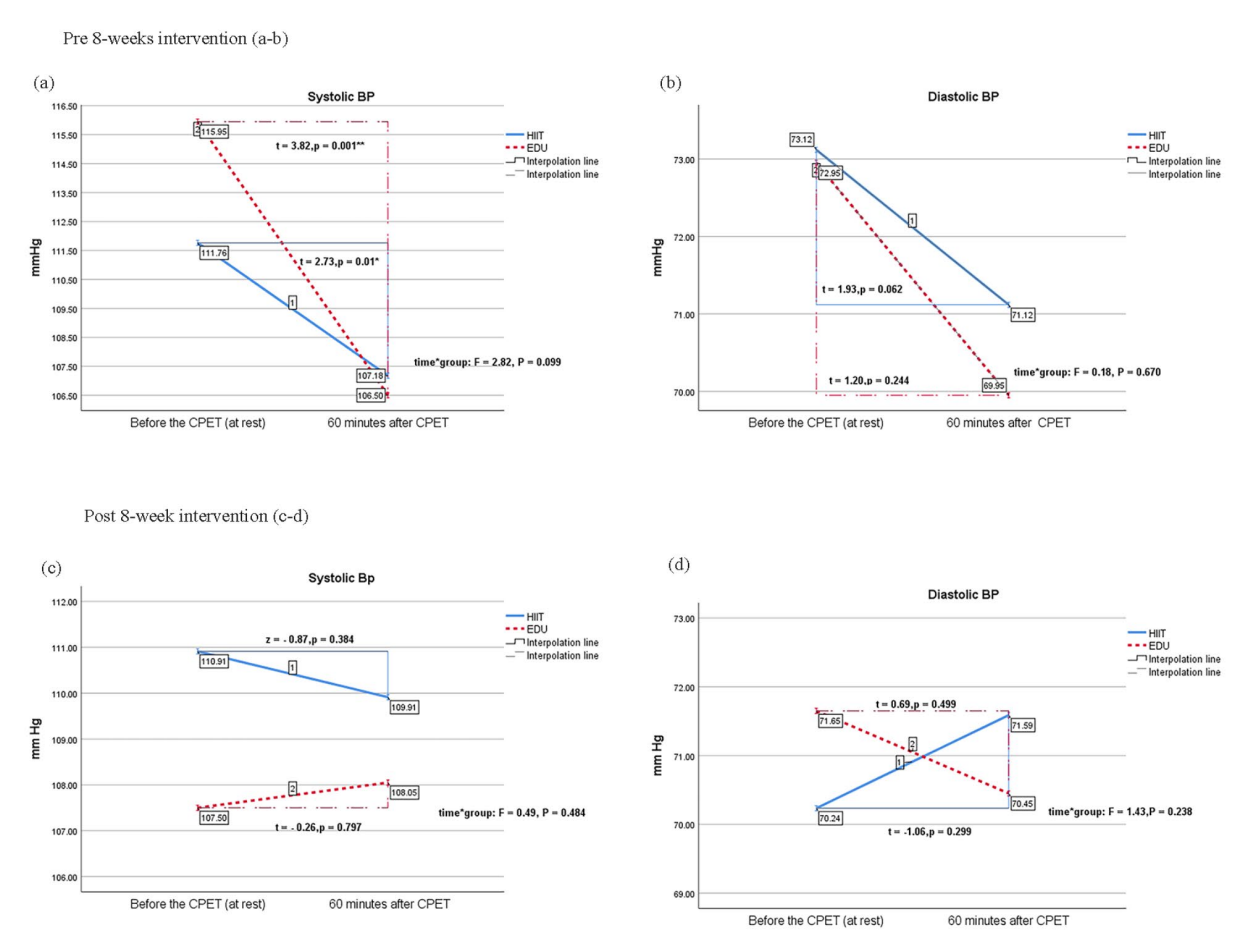
Changes in blood pressure before and 60 min after cardiorespiratory maximal progressive exercise testing. Box charts (**a**) and (**b**) show blood pressure changes during testing before the 8-week interventions. Box charts (c) and (d) show blood pressure changes during testing after the 8-week interventions. EDU: education, HIIT: high-intensity interval training, BP: blood pressure, CPET: cardiorespiratory maximal progressive exercise test, * *p* < 0.05, ** *p* < 0.01.

Before the 8-week interventions, both groups responded similarly to the CPET in terms of changes in systolic BP and diastolic BP (F = 2.82, *p* = 0.099, η^2^ = 0.05 and F = 0.18, *p* = 0.67, η^2^ = 0.004, respectively; Table 2). Based on independent analyses for both groups, the HIIT and EDU groups demonstrated a decrease in both systolic (Fig. 3a) and diastolic BP (Fig. 3b) from pre- to post-CPET, though only the change in systolic BP was statistically significant (HIIT: t = 2.73, *p* = 0.01 and EDU: t = 3.82, *p* = 0.001).

**Table 2:** Comparison of systolic and diastolic blood pressure before and 60 min after the intervention cardiorespiratory maximal progressive exercise test in the two groups pre the 8-week intervention.

Variable	Group	*N*	Time points		Time* group		
			Before CPET (at rest)	After CPET			
			(M ± SD)		F	*p*	ES
Systolic BP (mmHg)	HIIT	34	111.76 ± 9.42	107.18 ± 9.33	2.82	0.099	0.051
	EDU	20	115.95 ± 11.77	106.50 ± 10.73			
Diastolic BP (mmHg)	HIIT	34	73.12 ± 6.63	71.12 ± 7.26	0.18	0.670	0.004
	EDU	20	72.95 ± 8.66	69.95 ± 6.78			

EDU: education, HIIT: high-intensity interval training, CPET: cardiorespiratory maximal progressive exercise test, BP: blood pressure. M ± SD: mean ± standard deviation, F: Data were analyzed using repeated measure ANOVA, p: level of statistical significance, ES: effect size based on the partial η^2^ (small, η^2^ ≥ 0.01; medium, η^2^  ≥ 0.06 ; large, η^2^ ≥ 0.14).

After the 8-week interventions, still both groups responded to the CPET statistically similarly in terms of changes in systolic BP and diastolic BP (F = 0.49, *p* = 0.484, W = 0.01 and F = 0.143, *p* = 0.238, η^2^ = 0.03, respectively; Table 3). However, when analyzing the results of these two groups separately, we noticed an interesting trend: pre- to post-CPET, the HIIT and EDU groups presented opposite changes in systolic and diastolic BP. The HIIT group had a slight decrease while the EDU group had a slight increase in systolic BP (Fig. 3c). We observed the inverse trend for diastolic BP: the HIIT group had a slight increase while the EDU group had a slight decrease in diastolic BP (Fig. 3d). These changes were not statistically significant.

**Table 3:** Comparison of systolic and diastolic blood pressure before and 60 min after the intervention cardiorespiratory maximal progressive exercise test in the two groups post the 8-week intervention.

Variable	Group	*N*	Time points		Time* group		
			Before CPET (at rest)	After CPET			
			(M ± SD)		F	*p*	ES
Systolic BP (mmHg)	HIIT	34	110.91 ± 10.42	109.91 ± 12.40	0.49^b^	0.484	0.009^w^
	EDU	20	107.50 ± 10.91	108.05 ± 7.37			
Diastolic BP (mmHg)	HIIT	34	70.24 ± 8.37	71.59 ± 6.95	1.43^a^	0.238	0.027
	EDU	20	71.65 ± 8.44	70.45 ± 9.04			

EDU: education, HIIT: high-intensity interval training, CPET: cardiorespiratory maximal progressive exercise test, BP: blood pressure. M ± SD: mean ± standard deviation, F: data were analyzed using repeated measure ANOVA (a) or Friedman test (b), p: level of statistical significance, ES: effect size based on the partial η^2^ (small, η^2^ ≥ 0.01; medium, η^2^  ≥ 0.06 ; large, η^2^ ≥ 0.14) or W: Kendall’s W.

#### Comparative analysis of cardiopulmonary parameters pre- and post-intervention

**Fig. 4:**
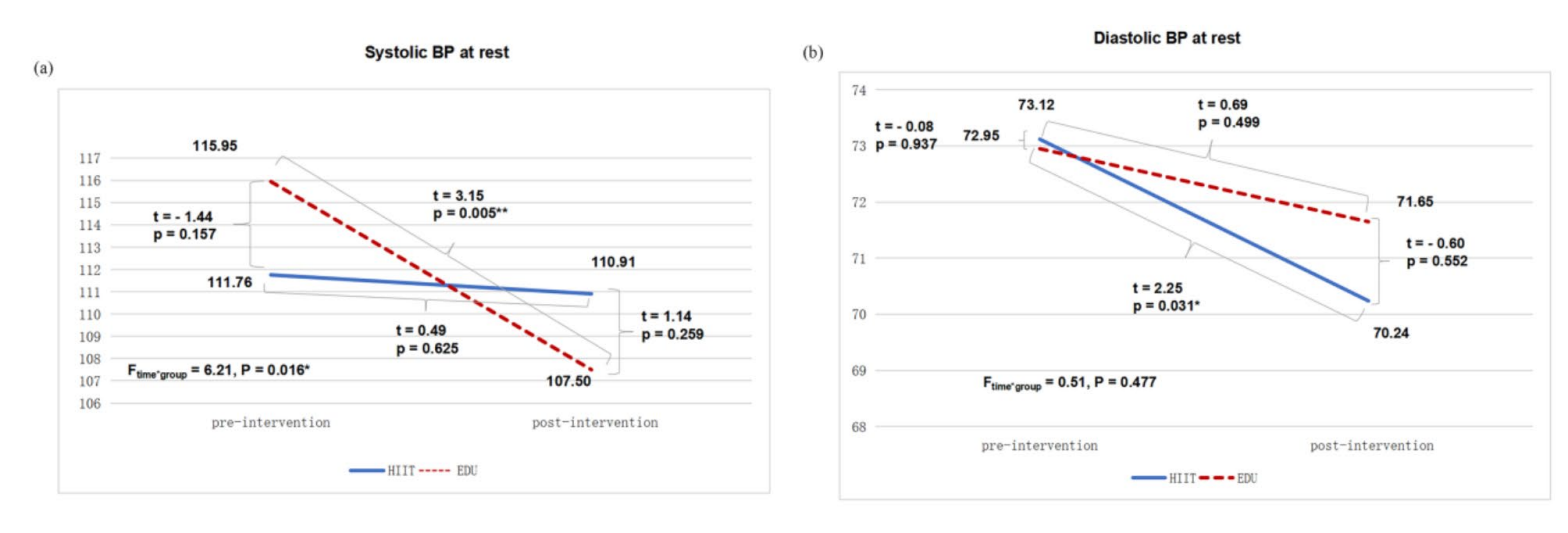
Differences in BP between high-intensity interval training and education groups pre‑ and post 8-weekintervention. (**a**) Systolic BP at rest; (**b**) diastolic BP at rest. BP: blood pressure, EDU: education, HIIT: high-intensity interval training, p: level of statistical significance, * *p* < 0.05, ** *p* < 0.01.

When assessing changes in resting BP before and after the 8-week interventions, we found a statistically significant difference between groups in systolic BP (F = 6.21, *p* = 0.016, η^2^ = 0.11, medium effect). We observed a significant decrease in systolic BP in the EDU group (*p* = 0.005). In the HIIT group, the systolic BP remained at similar level after 8 weeks (Fig. 4a). We didn’t note statistical differences between groups in the effectiveness of HIIT or EDU interventions on diastolic BP (F = 0.51, *p* = 0.47, η^2^ = 0.01), however in both groups the values of this parameter showed a decreasing trend after the intervention (Fig. 4b). In an independent analysis of pre- and post-intervention differences for individual groups, this decrease was statistically significant only in the HIIT group (*p* = 0.031).

Importantly, after 8 weeks, we found statistically significant differences in the effects of the interventions between groups in terms of VO_2peak_ (F = 23.13, *p* < 0.001, η^2^ = 0.31) and HR_max_ (F = 10.40, *p* = 0.002, η^2^ = 0.17; Table 4). Despite the progression of pregnancy and the associated increased body weight, the HIIT group maintained the values of VO_2peak_ (Pre: 25.71 ± 4.29 and post 25.67 ± 4.52, *p* = 0.943) and HR_max_ (Pre: 167.32 ± 10.07, post: 166.50 ± 10.73, *p* = 0.527) at the similar level. Whereas, the EDU group presented significantly worse outcomes in these cardiorespiratory parameters comparing to baseline (VO_2peak_: Pre 23.69 ± 3.58 and post 19.75 ± 4.1, *p* < 0.001), and HR_max_ (Pre: 166.05 ± 12.54, post: 155.00 ± 15.43, *p* < 0.001).

**Table 4:** Comparison of cardiopulmonary parameters pre- and post the 8-week intervention.

Variable	Group	*N*	Time points		Time* group		
			Pre-intervention (at rest)	Post-intervention (at rest)			
			(M ± SD)		F	*p*	ES
Systolic BP at rest (mmHg)	HIIT	34	111.76 ± 9.42	110.91 ± 10.42	6.21	0.016*	0.107
	EDU	20	115.95 ± 11.77	107.50 ± 11.91			
Diastolic BP at rest (mmHg)	HIIT	34	73.12 ± 6.63	70.24 ± 8.37	0.51	0.477	0.010
	EDU	20	72.95 ± 8.66	71.65 ± 8.44			
HR at rest (beat min^− 1^)	HIIT	34	82.00 ± 10.11	81.94 ± 11.54	4.95	0.030*	0.087
	EDU	20	87.70 ± 15.77	81.75 ± 15.01			
VO_2peak_ (mL/kg/min)	HIIT	34	25.71 ± 4.29	25.67 ± 4.52	18.81	0.000**	0.266
	EDU	20	23.69 ± 3.58	19.75 ± 4.11			
HR_max_ (beat min^− 1^)	HIIT	34	167.32 ± 10.07	166.50 ± 10.73	15.59	0.000**	0.231
	EDU	20	166.05 ± 12.54	155.00 ± 15.43			

EDU: education, HIIT: high-intensity interval training, HRmax: maximum heart rate, VO_2peak_: maximal oxygen uptake, BP: blood pressure. M ± SD: mean ± standard deviation, F: data were analyzed using repeated measure ANOVA, p: level of statistical significance. * *p* < 0.05, ** *p* < 0.01, ES: effect size based on the partial η^2^ (small, η^2^ ≥ 0.01; medium, η^2^  ≥ 0.06 ; large, η^2^ ≥ 0.14).

### Relationship between blood pressure and weight, parity, body mass index, and cardiorespiratory fitness

**Fig. 5:**
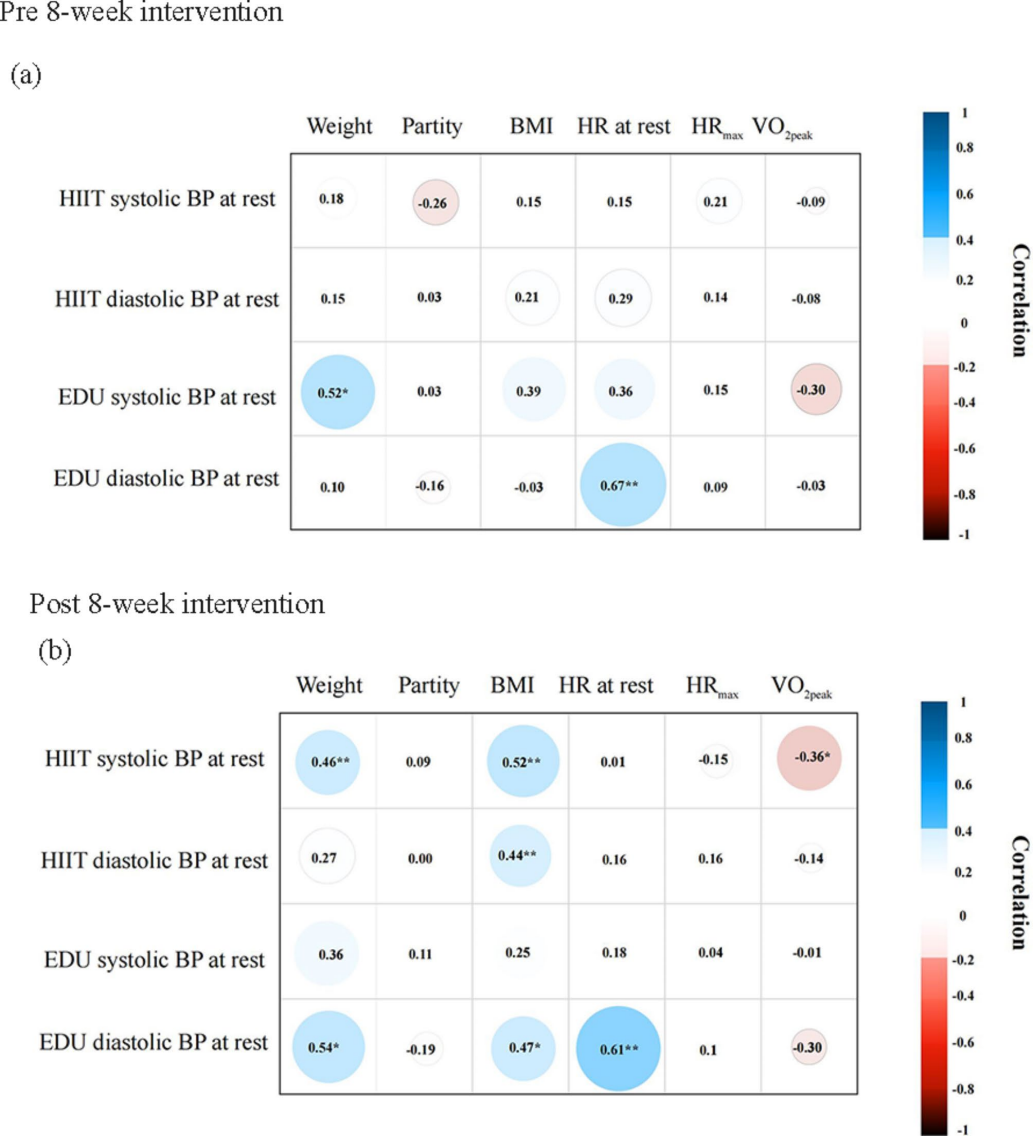
Heat map of Pearson correlation coefficients between BP and weight, parity, BMI, and cardiorespiratory fitness pre- and post-8 weeks intervention in the high-intensity interval training and education groups. BMI: body mass index, BP: blood pressure, EDU: education, HIIT: high-intensity interval training, HR: heart rate, VO_2peak_: maximal oxygen uptake, p: level of statistical significance, * *p* < 0.05, ** *p* < 0.01.

Pearson’s correlation coefficients between systolic and diastolic BP at rest and weight, parity, BMI, and cardiopulmonary parameters (HR at rest, HR_max_, VO_2peak_) were calculated for the two time points: before and after 8-week interventions (Fig. 5). At the pre-intervention time point, in the EDU group weight was positively correlated with systolic BP at rest (*r* = 0.52, *p* < 0.05) and HR at rest was positively correlated with diastolic BP at rest (*r* = 0.36, *p* < 0.01).

After 8-week interventions, in the HIIT group weight (*r* = 0.46, *p* < 0.01) and BMI (*r* = 0.52, *p* < 0.01) were positively correlated with systolic BP at rest, while VO_2peak_ was negatively correlated with systolic BP at rest (*r* = -0.36, *p* < 0.05). BMI was also positively correlated with diastolic BP (*r* = 0.44, *p* < 0.05). In the EDU group weight (*r* = 0.54, *p* < 0.05), BMI (*r* = 0.47, *p* < 0.05), and HR at rest (*r* = 0.61, *p* < 0.01) were positively correlated with diastolic BP.

## Discussion

In this study, we compared an 8-week HIIT program to self-performed moderate-to-vigorous PA during pregnancy by evaluating pre- and post-intervention changes in BP before and after a maximal exercise test. To our knowledge, this is the first study to assess the efficacy of HIIT program in regulating BP during pregnancy. One of our most important findings was that after 8 weeks of either intervention, supervised HIIT or self-performed moderate-to-vigorous PA, pregnant women maintained normal BP values. No cases of hypertension were observed in either group. HIIT can thus be recommended as an effective exercise mode to prevent gestational hypertension, similar to moderate-to-vigorous PA, which is currently a common recommendation for pregnant women.

In previous research, relative BP response to exercise has been found to be inversely related to cardiorespiratory fitness measured at the outset of the second trimester of pregnancy, suggesting that regular aerobic exercise can attenuate exaggerated BP responses to exercise in pregnant women^39^. Similarly, in the current study, both the HIIT and EDU groups demonstrated a significant post-CPET decrease in diastolic BP at baseline, but not after completion of the interventions. This may indicate that 8 weeks of HIIT or moderate-to-vigorous PA interventions elicit acute exercise adaptations that include BP adaptations to maximal physical exertion, with the added benefit of causing no adverse reactions among participants.

The results of a previous meta-analysis identified that physical exercise during pregnancy can effectively reduce BP^40^. Similarly, in a study evaluating a 12-week exercise intervention among healthy and nonactive pregnant women, changes in resting BP from baseline to post-intervention were assessed, and the researchers found that regular, long-term physical exercise significantly reduced resting systolic BP^20^. One of the meta-analysis further revealed that physical exercise interventions during pregnancy reduced the risk of pregnancy-induced hypertension and preeclampsia by 39% and 41%, respectively^41^. Consistent with these findings, following the 8-week interventions implemented in the current study, both systolic and diastolic BP substantially decreased or tended to decrease in the HIIT and EDU groups, remaining within normal ranges. Notably, the EDU group exhibited a greater reduction in systolic BP compared to the HIIT group. In the EDU group, the post-intervention systolic blood pressure of 107.5 mmHg was slightly lower than the recommended level of 110 mmHg. However, this difference does not appear to have clinical significance. Several women presented BP values below 110/65 mmHg. Nevertheless, based on additional questions related to their well-being, we still classified them as normotensive participants^16^. Our findings suggest that moderate-to-vigorous exercise may be more beneficial for hypertensive women than HIIT in terms of high systolic BP. On the other hand, HIIT can be more effective to normalize diastolic BP. Further investigation is required to test this hypothesis. What is more, our results may indicate the need to verify reference values for the lower BP ranges for active, healthy pregnant women.

Interestingly, post 8 week-interventions the HIIT and EDU groups showed opposite changes in BP after maximal physical exertion. After CPET the HIIT group had a slight decrease while the EDU group had a slight increase in systolic BP, and the HIIT group had a slight increase while the EDU group had a slight decrease in diastolic BP. Our data indicate the need for further research to determine what type of exercise and what intensity will be most beneficial for women at risk of blood pressure disorders during pregnancy. Studies should include groups of pregnant women with both hypertension and hypotension.

We observed that HIIT, apart from maintaining normotension, also improves the cardiorespiratory fitness of pregnant women. The measures employed in this study to reflect exercise capacity, VO_2peak_ and HR_max_, exhibited a significant decrease in the EDU group post-intervention compared to pre-intervention while remaining stable in the HIIT group. In our previous studies we discussed the issue, that the HIIT group maintained VO_2peak_, HR/AT and VO2/AT levels after intervention and that the values of these parameters were significantly higher than those in EDU group^42,43^. Therefore, HIIT can be considered an effective strategy for preventing pregnancy-related declines in exercise capacity. In addition, findings from other studies suggest that a combination of aerobic and anaerobic exercise or yoga may offer greater benefits than aerobic exercise alone in reducing the risk of hypertensive disorders of pregnancy^44^.

Finally, to better study BP during pregnancy, we analyzed the relationships between BP and parity, BMI, and cardiorespiratory fitness (HR, HR_max_, and VO_2peak_) in the HIIT and EDU groups pre- and post-intervention. A previous study has suggested a potential influence of maternal parity on BP levels^45^; however, no significant correlation was found between parity and BP in either the HIIT or EDU groups in the current study. Additionally, some studies have shown an increase in mean BP with increasing BMI^46^, though PA may still improve BP status during pregnancy through indirect effects on the management of BMI and/or weight gain^47^. Consistent with these previous findings, the current study revealed a positive correlation between BMI and BP in the HIIT and EDU groups post-intervention. This study further showed that VO_2peak_ was negatively correlated with BP, with a significant negative correlation found between VO_2peak_ and systolic BP in the HIIT group. This agrees with previous research showing that relative BP response to exercise is inversely related to cardiorespiratory fitness measured at the second trimester of pregnancy and associated with higher levels of fitness^39^. As there is limited data on the effect of regular exercise on resting BP in pregnant women, correlations between BP and weight, parity, BMI, and cardiorespiratory fitness (HR, HR_max_, and VO_2peak_) require further research.

There is a very low level of PA during pregnancy in different populations worldwide^48^. Referring to our previous systematic review of factors influencing PA during pregnancy based on socio-ecological model, reasons for lack of PA among pregnant women include lack of time, safety concerns, lack of information and resources, and lack of facilities^49^. It is thus necessary to promote initiatives and exercise programs that will help pregnant women to overcome these barriers and provide accessible information and resource systems for pregnant women. Previous studies and this study have shown that providing exercise information and behavioral interventions to pregnant women can help them maintain normal BP^50–52^. While, a study that highlighted poor adherence to World Health Organization recommendations for aerobic activity or moderate-intensity continuous training protocols prompted searches for alternative exercise modes to address continuing insufficient PA levels^22^. Given the results of the current study, HIIT may constitute an effective alternative exercise strategy during pregnancy, as, compared to the EDU group, the HIIT group demonstrated better adherence and a relatively low dropout rate. Furthermore, according to Tamayo Acosta et al., HIIT offers similar benefits as low- to moderate-intensity aerobic exercise in terms of BP regulation while also being more effective and efficient in increasing cardiorespiratory fitness observed in higher VO_2peak_ values^23^. The findings of the current study provide support for the application of HIIT interventions during pregnancy, and we recommend HIIT as an alternative exercise prescription for pregnant women. We need to emphasize that the provision of effective information to pregnant women, such as PA guidelines on exercise during pregnancy, is fundamental to these interventions. For pregnant women themselves, PA or exercise prescriptions tailored individually by appropriate specialists or trained clinicians may be the most effective means to help them meet PA recommendations^49^. We believe that it is necessary to integrate activities promoting PA during pregnancy into the health care systems.

This study has limitations that should be noted. First, some participants dropped out of the study due to interference from external factors, only completed part of the intervention, or did not strictly adhere to the study’s protocols. Secondly, we included only healthy, normotensive women in a single pregnancy in the study. It would be valuable for future research to repeat the same experiment in different population of pregnant women, including patients with BP disorders and women with multiple pregnancies. An additional limitation of our study is its lack of nutrition or energy intake assessment; however, all participants received standard care and information regarding a healthy lifestyle during pregnancy. Finally, the lack of data collection on family history of hypertension was a limitation of the study’s methodology. An interesting idea is to add non-pregnant women to the experiment as an additional comparison group. Despite these limitations, this study is the first to evaluate the effects of a supervised HIIT program on BP-related parameters in pregnant women, providing new ideas for reliable and safe strategies for maintaining normal BP levels during pregnancy.

## Conclusion

Throughout the 8-week supervised HIIT and self-performed moderate-to-vigorous intensity interventions pregnant women maintain normal values of BP. After eight weeks, we observed an adaptive change in lower BP lability after maximal physical exertion. This may indicate an improvement in the effectiveness of the acute regenerative processes of the cardiopulmonary system after exercise interventions. It seems that both supervised HIIT and self-performed moderate-to-vigorous PA can be recommended as strategies to prevent BP disorders during pregnancy. However, to maintain or improve cardiorespiratory fitness, pregnant women need exercise of higher intensity. More studies are needed to confirm our findings.

## Electronic supplementary material

Below is the link to the electronic supplementary material.

Supplementary Material 1

## Data Availability

All data generated or analysed during this study are included in this published article [and its supplementary information files].

## References

[1] Weissgerber, T. L. & Wolfe, L. A. Physiological adaptation in early human pregnancy: Adaptation to balance maternal-fetal demands. *Appl. Physiol. Nutr. Metab.* **31**, 1–11 (2006). 16604136 10.1139/h05-003

[2] Gregg, V. H. & Ferguson, J. E. Exercise in pregnancy. *Clin. Sports Med.* **36**, 741–752 (2017). 28886825 10.1016/j.csm.2017.05.005

[3] Eshriqui, I. et al. Gestational dietary patterns are not associated with blood pressure changes during pregnancy and early postpartum in a Brazilian prospective cohort. *Eur. J. Nutr.* **55**, 21–32 (2016). 25526968 10.1007/s00394-014-0819-4

[4] Hermida, R. C., Ayala, D. E. & Iglesias, M. Predictable blood pressure variability in healthy and complicated pregnancies. *Hypertension*. **38**, 736–741 (2001). 11566967 10.1161/01.hyp.38.3.736

[5] Loerup, L. et al. Trends of blood pressure and heart rate in normal pregnancies: A systematic review and meta-analysis. *BMC Med.* **17**, 167 (2019). 31506067 10.1186/s12916-019-1399-1 PMC6737610

[6] de Haas, S. et al. Blood pressure adjustments throughout healthy and hypertensive pregnancy: A systematic review and meta-analysis. *Pregnancy Hypertens.* **27**, 51–58 (2022). 34929556 10.1016/j.preghy.2021.12.004

[7] Sanghavi, M. & Rutherford, J. D. Cardiovascular physiology of pregnancy. *Circulation*. **130**, 1003–1008 (2014). 25223771 10.1161/CIRCULATIONAHA.114.009029

[8] Duley, L. The global impact of pre-eclampsia and eclampsia. *Semin. Perinatol*. **33**, 130–137 (2009). 19464502 10.1053/j.semperi.2009.02.010

[9] Steegers, E. A., von Dadelszen, P., Duvekot, J. J. & Pijnenborg, R. Pre-eclampsia. *Lancet*. **376**, 631–644 (2010). 20598363 10.1016/S0140-6736(10)60279-6

[10] Abalos, E., Cuesta, C., Grosso, A. L., Chou, D. & Say, L. Global and regional estimates of preeclampsia and eclampsia: A systematic review. *Eur. J. Obstet. Gynecol. Reprod. Biol.* **170**, 1–7 (2013). 23746796 10.1016/j.ejogrb.2013.05.005

[11] NICE Guideline. Hypertension in pregnancy: Diagnosis and management Accessed 20 February, 2023. (2019).

[12] Webster, L. M. et al. Impact of Antihypertensive treatment on maternal and perinatal outcomes in pregnancy complicated by chronic hypertension: A systematic review and Meta-analysis. *J. Am. Heart Assoc.* **6**, e005526 (2017). 28515115 10.1161/JAHA.117.005526 PMC5524099

[13] Yeh, P. T. et al. Self-monitoring of blood pressure among women with hypertensive disorders of pregnancy: A systematic review. *BMC Pregnancy Childbirth*. **22**, 454 (2022). 35641913 10.1186/s12884-022-04751-7 PMC9152837

[14] Friedman, E. A. Hypertension-hypotension in pregnancy: Correlation with fetal outcome. *JAMA*. **239**, 2249 (1978). 650804

[15] Davies, G. A. L., Wolfe, L. A., Mottola, M. F. & MacKinnon, C. Joint SOGC/CSEP clinical practice guideline: Exercise in pregnancy and the postpartum period. *Can. J. Appl. Physiol.* **28**, 329–341 (2003). 12955862

[16] Wowdzia, J. B. & Davenport, M. H. Cardiopulmonary exercise testing during pregnancy. *Birth Defects Res.* **113**, 248–264 (2021). 32894003 10.1002/bdr2.1796

[17] Yeo, S. Prenatal stretching Exercise and autonomic responses: Preliminary Data and a model for reducing Preeclampsia: Prenatal stretching Exercise and Preeclampsia. *J. Nurs. Scholarsh.* **42**, 113–121 (2010). 20618595 10.1111/j.1547-5069.2010.01344.x PMC2904621

[18] Barakat, R. et al. Exercise during pregnancy protects against hypertension and macrosomia: Randomized clinical trial. *Am. J. Obstet. Gynecol.* **214**, 649e1–649e8 (2016). 10.1016/j.ajog.2015.11.039 26704894

[19] Falcao, S. et al. Exercise training can attenuate preeclampsia-like features in an animal model. *J. Hypertens.* **28**, 2446–2453 (2010). 20811291 10.1097/HJH.0b013e32833e97d0

[20] Haakstad, L. A. H., Edvardsen, E. & Bø, K. Effect of regular exercise on blood pressure in normotensive pregnant women. A randomized controlled trial. *Hypertens. Pregnancy*. **35**, 170–180 (2016). 26909888 10.3109/10641955.2015.1122036

[21] Bull, F. C. et al. World Health Organization 2020 guidelines on physical activity and sedentary behaviour. *Br. J. Sports Med.* **54**, 1451–1462 (2020). 33239350 10.1136/bjsports-2020-102955 PMC7719906

[22] Wood, G., Murrell, A., van der Touw, T. & Smart, N. HIIT is not superior to MICT in altering blood lipids: A systematic review and meta-analysis. *BMJ Open. Sport Exerc. Med.* **5**, e000647 (2019). 31921439 10.1136/bmjsem-2019-000647 PMC6937112

[23] Tamayo Acosta, J. *et al*. Effects of aerobic exercise versus high-intensity interval training on V̇O2max and blood pressure. *Cureus* **14**, e30322 (2022). 36407200 10.7759/cureus.30322 PMC9661924

[24] Szumilewicz, A. et al. How to HIIT while pregnant? The protocol characteristics and effects of high intensity interval training implemented during pregnancy – a systematic review. *Balt J. Health Phys. Activity*. **14**, 1–16 (2022).

[25] Szumilewicz, A. *et al.* Translation and cross-cultural adaptation of the Get Active Questionnaire for Pregnancy (kwestionariusz “Badź Aktywna w Ciąży”) to support physical activity among pregnant women in Poland. *Ginekol. Pol*. **95**, 483–501 (2024). 10.5603/gpl.97888 38334341

[26] Nosek, B. A. et al. Promoting an open research culture. *Science*. **348**, 1422–1425 (2015). 26113702 10.1126/science.aab2374 PMC4550299

[27] Craig, C. L. et al. International physical activity questionnaire: 12-Country reliability and validity. *Med. Sci. Sports Exerc.* **35**, 1381–1395 (2003). 12900694 10.1249/01.MSS.0000078924.61453.FB

[28] IPAQ Research Committee. Guidelines for data processing and analysis of the international physical activity questionnaire (ipaq)-short and long forms. *UK Biobank* https://biobank.ndph.ox.ac.uk/showcase/ukb/docs/ipaq_analysis.pdf (2005).

[29] Cheng, H. L. A simple, easy-to-use spreadsheet for automatic scoring of the international physical activity questionnaire (IPAQ) short form. *ResearchGate* (2016).

[30] Ross, R. M. ATS/ACCP statement on cardiopulmonary exercise testing. *Am. J. Respir Crit. Care Med.* **167**, 1451–1451 (2003). 12738602 10.1164/ajrccm.167.10.950

[31] Walker, H. K., Hall, W. D. & Hurst, J. W. (eds) in *Clinical Methods: The History, Physical, and Laboratory Examinations* 3rd edn. (Butterworths, 1990). 21250045

[32] Moser, M. Working group report on high blood pressure in pregnancy. *J. Clin. Hypertens.* **3**, 75–88 (2001). 10.1111/j.1524-6175.2001.00458.x PMC8099347 11416689

[33] Santos-Rocha, R., De Carvalho, F., De Freitas, M. P., Wegrzyk, J., Szumilewicz, A. & J. & Active pregnancy: A physical Exercise Program promoting fitness and health during pregnancy—development and validation of a complex intervention. *Int. J. Environ. Res. Public. Health*. **19**, 4902 (2022). 35457769 10.3390/ijerph19084902 PMC9028999

[34] Kwiatkowska, E. *et al.* Polish Society of Gynecologists and Obstetricians (PTGiP) and Polish Society of Sports Medicine (PTMS) recommendations on physical activity during pregnancy and the postpartum period. *Ginekol. Pol*. **95**, 218–231 (2024). 10.5603/GP.a2023.0080 37599577

[35] Beaver, W. L., Wasserman, K. & Whipp, B. J. A new method for detecting anaerobic threshold by gas exchange. *J. Appl. Physiol.* **60**, 2020–2027 (1986). 3087938 10.1152/jappl.1986.60.6.2020

[36] Borg, G. *Borg’s Perceived Exertion And Pain Scales*. (1998).

[37] Persinger, R., Foster, C., Gibson, M., Fater, D. C. & Porcari, J. P. Consistency of the talk test for exercise prescription. *Med. Sci. Sports Exerc.* **36**, 1632–1636 (2004). 15354048

[38] Szumilewicz, A. & Santos-Rocha, R. Exercise selection and adaptations during pregnancy. In *Exercise and Physical Activity During Pregnancy and Postpartum Evidence-Based Guidelines* (ed. Santos-Rocha, R.) 275–361 (Springer, 2022).

[39] Bisson, M., Rhéaume, C., Bujold, E., Tremblay, A. & Marc, I. Modulation of blood pressure response to exercise by physical activity and relationship with resting blood pressure during pregnancy. *J. Hypertens.* **32**, 1450–1457 (2014). 24721929 10.1097/HJH.0000000000000185

[40] Zhu, Z. et al. Effects of physical exercise on blood pressure during pregnancy. *BMC Public. Health*. **22**, 1733 (2022). 36096756 10.1186/s12889-022-14074-z PMC9469521

[41] Davenport, M. H. et al. Prenatal exercise for the prevention of gestational diabetes mellitus and hypertensive disorders of pregnancy: A systematic review and meta-analysis. *Br. J. Sports Med.* **52**, 1367–1375 (2018). 30337463 10.1136/bjsports-2018-099355

[42] Wilczyńska, D. et al. Can we hit prenatal depression and anxiety through HIIT? The effectiveness of online high intensity interval training in pregnant women during the COVID-19 pandemic: A randomized controlled trial. *BMC Sports Sci. Med. Rehabil*. **14**, 215 (2022). 36550564 10.1186/s13102-022-00610-2 PMC9773485

[43] Yu, H. et al. Effects of 8-week online, supervised high-intensity interval training on the parameters related to the anaerobic threshold, body weight, and body composition during pregnancy: A randomized controlled trial. *Nutrients*. **14**, 5279 (2022). 36558438 10.3390/nu14245279 PMC9781372

[44] Khunti, K. & Tan, B. Effects of supervised exercise on the development of hypertensive disorders of pregnancy: A systematic review and Meta-analysis. *J. Clin. Med.* **11**, 793 (2022). 35160245 10.3390/jcm11030793 PMC8836524

[45] Tejera, E., Areias, M. J., Rodrigues, A. I., Nieto-Villar, J. M. & Rebelo, I. Blood pressure and heart rate variability complexity analysis in pregnant women with hypertension. *Hypertens. Pregnancy*. **31**, 91–106 (2012). 21599453 10.3109/10641955.2010.544801

[46] Thompson, M. L., Williams, M. A. & Miller, R. S. Modelling the association of blood pressure during pregnancy with gestational age and body mass index. *Paediatr. Perinat. Epidemiol.* **23**, 254–263 (2009). 19775387 10.1111/j.1365-3016.2009.01027.x

[47] Thangaratinam, S. et al. Effects of interventions in pregnancy on maternal weight and obstetric outcomes: Meta-analysis of randomised evidence. *BMJ*. **344**, e2088–e2088 (2012). 22596383 10.1136/bmj.e2088 PMC3355191

[48] Silva-Jose, C., Sánchez-Polán, M., Barakat, R., Gil-Ares, J. & Refoyo, I. Level of physical activity in pregnant populations from different Geographic regions: A systematic review. *J. Clin. Med.* **11**, 4638 (2022). 35956253 10.3390/jcm11154638 PMC9369818

[49] Sun, J., Piernicka, M., Worska, A. & Szumilewicz, A. A socio-ecological model of factors influencing physical activity in pregnant women: A systematic review. *Front. Public. Health*. **11**, 1232625 (2023). 38054068 10.3389/fpubh.2023.1232625 PMC10694207

[50] Barakat, R., Perales, M., Bacchi, M., Coteron, J. & Refoyo, I. A program of exercise throughout pregnancy. Is it safe to mother and newborn? *Am. J. Health Promot.* **29**, 2–8 (2014). 24200335 10.4278/ajhp.130131-QUAN-56

[51] Petrov Fieril, K., Glantz, A. & Fagevik Olsen, M. The efficacy of moderate-to‐vigorous resistance exercise during pregnancy: A randomized controlled trial. *Acta Obstet. Gynecol. Scand.* **94**, 35–42 (2015). 25287282 10.1111/aogs.12525

[52] Silva-Jose, C. et al. Effectiveness of a virtual exercise program during COVID-19 confinement on blood pressure control in healthy pregnant women. *Front. Physiol.* **12**, 645136 (2021). 33776798 10.3389/fphys.2021.645136 PMC7988209

